# Facile Fabrication of Flower-Like BiOI/BiOCOOH p–n Heterojunctions for Highly Efficient Visible-Light-Driven Photocatalytic Removal of Harmful Antibiotics

**DOI:** 10.3390/nano9111571

**Published:** 2019-11-06

**Authors:** Shijie Li, Bing Xue, Chunchun Wang, Wei Jiang, Shiwei Hu, Yanping Liu, Hengwei Wang, Jianshe Liu

**Affiliations:** 1 Key Laboratory of Health Risk Factors for Seafood of Zhejiang Province, Institute of Innovation & Application, Zhejiang Ocean University, Zhoushan 316022, China; lishijie@zjou.edu.cn (S.L.); xb1725621827@163.com (B.X.); liuyp@zjou.edu.cn (Y.L.);; 2 College of Marine Science and Technology, Zhejiang Ocean University, Zhoushan 316022, China; 3 State Environmental Protection Engineering Center for Pollution Treatment and Control in Textile Industry, College of Environmental Science and Engineering, Donghua University, Shanghai 201620, China; liujianshe@dhu.edu.cn

**Keywords:** flower-like heterostructure, BiOI/BiOCOOH, p–n heterojunction, visible light photocatalysis, antibiotic removal

## Abstract

Novel heterojunction photocatalysts with remarkable photocatalytic capabilities and durability for degrading recalcitrant contaminants are extremely desired; however, their development still remains quite challenging. In this study, a series of flower-like BiOI/BiOCOOH p–n heterojunctions were fabricated via a controlled in situ anion-exchange process. During the process, BiOI formation and even deposition on BiOCOOH microspheres with tight interfacial contact were realized. As expected, BiOI/BiOCOOH heterojunctions revealed remarkable enhancements in photocatalytic antibiotic degradation capacities under visible light irradiation compared with pristine BiOI and BiOCOOH. The best-performing BiOI/BiOCOOH heterojunction (i.e., IBOCH-2) showed much improved photocatalytic CIP degradation efficiency of approximately 81- and 3.9-fold greater than those of bare BiOI and BiOCOOH, respectively. The eminent photocatalytic performances were due not only to the enhanced capability in harvesting photon energies in visible light regions, but also the accelerated separation of electrons and holes boosted by the p–n heterojunction. Active species trapping tests demonstrated that superoxide free radicals (•O_2_^−^) and photo-generated holes (h^+^) were major active species for CIP degradation. Recycling experiments verified the good durability of BIBO-2 over four runs. The facile in situ synthesis route and excellent performance endow flower-like BiOI/BiOCOOH heterojunctions with a promising potential for actual environmental remediation.

## 1. Introduction

In recent decades, the widespread occurrence of pharmaceutical antibiotics in the environment has induced overwhelming concerns for human health. Thus, searching for a viable approach to efficiently eliminate these harmful antibiotics is an urgent issue. Semiconductor-based photocatalysis as an environmentally friendly and high-efficiency treatment represents a promising method to protect and remedy the environment [1,2,3,4]. Nowadays, bismuth-based semiconductors have evoked great interest for their exclusive electronic and structural characteristics [5,6,7,8,9,10,11]. Typically, by virtue of its unique layered architecture, fast charge separation, strong redox ability, and good chemical stability, n-type BiOCOOH is demonstrated to be an active photocatalyst for toxic contaminant removal [12,13]. Nevertheless, the severe recombination of photo-excited electron-hole pairs and the insufficient utilization of sunlight (merely 4% of solar energy) have substantially restrained its further application [14,15,16,17,18]. The fabrication of heterojunctions is an effective strategy to upgrade photocatalytic behavior by remarkably boosting the separation efficiency and/or substantially extending the optical absorption range. Developing novel BiOCOOH-based heterojunctions with extraordinary catalytic behavior is very imperative but still a huge challenge.

P-type bismuth oxyiodide (BiOI) has been widely applied in the photocatalytic treatment of wastewater because of its excellent light absorption characteristics (*Eg* = ∼1.8 eV) and high electron-hole separation rate [19,20,21,22]. To date, numerous studies have shown that the integration of BiOI with semiconductor materials can pronouncedly ameliorate the photocatalytic performance. For instance, BiOI/Bi_2_O_2_CO_3_ [23], BiOI/BiOCl [24], BiOI/Bi_12_O_17_Cl_2_ [25], BiOI/SnO_2_ [26], and BiOI/BN [27] all exhibited superior activity compared to the constituents. Hence, it could be a fantasy strategy to upgrade the photocatalytic performance of BiOCOOH by fabricating BiOI/BiOCOOH p–n heterojunctions. Moreover, due to the strong interaction between the architecture of the semiconductor and photocatalytic performance, it is extremely desirable and promising to fabricate 3D flower-like BiOI/BiOCOOH p–n heterojunctions that are endowed with easily recycling characteristics and remarkable photocatalytic performance.

Inspired by these intriguing ideas, flower-like BiOI/BiOCOOH p–n heterojunctions were developed via a facile route, where BiOI was in situ anchored on BiOCOOH microspheres. These heterojunctions displayed superior photocatalytic behavior for antibiotic (CIP and TC) elimination. Furthermore, the photoluminescence (PL) spectra were measured to illustrate the interfacial charge separation of BiOI/BiOCOOH. More importantly, this study could provide enlightenment for developing 3D BiOCOOH-based heterojunction photocatalysts with admirable photocatalytic performance.

## 2. Experiment 

### 2.1. Reagents

All reagents of analytical grade were obtained from Chinese Sinopharm (Shanghai, China).

### 2.2. Fabrication of Photocatalysts 

Flower-like BiOCOOH microspheres were synthesized referring to a previous route [13,28]. BiOI/BiOCOOH heterojunctions were synthesized through a controlled in situ anion-exchange method. Firstly, 2 mmol/L of BiOCOOH powder was uniformly dispersed into 70 mL of KI (X mmol/L) solution in a beaker under constant stirring for 1 h while the pH of the suspension was maintained at pH 4.5. Subsequently, the suspension was placed in a 100 mL autoclave and heated at 140 °C for 15 h. After repeatedly washing 3 times, the product was collected after dryness. For convenience, these as-fabricated BiOI/BiOCOOH heterojunctions were labeled as IBOCH-X, where X refers to the amount of KI added as either 0.1, 0.2, 0.3, or 0.5 mmol/L.

### 2.3. Characterization

The characterization methods are shown in the supporting information (i.e., Experimental Section).

### 2.4. Photocatalytic Tests

Batch tests of CIP and TC elimination were implemented under visible light illumination to assess the catalytic behavior of the as-fabricated catalysts. Visible light, with a light intensity of ∼4.89 KW/m^2^ measured by an optical radiometer, was produced by a 300 W Xe lamp with a 400 nm cutoff glass filter. The distance between the surface of reaction solution and the light source was about 20 cm. Then, photocatalyst powder (35 mg) was dispersed into a CIP (10 mg/L, 80 mL, pH = 6.8) or TC (15 mg/L, 80 mL, pH = 6.4) aqueous solution. During the reaction, samples were taken at 25 min intervals, and the concentration of CIP or TC was analyzed using an UV-2600 spectrophotometer. To conduct the recycling experiments, the IBOCH-2 sample collected after each round was washed thoroughly with water and dried at 70 °C overnight for the subsequent run. On account of the inevitable loss of catalysts during the recycling process, several parallel runs under identical conditions were also performed to replenish the photocatalyst and ensure that the amount of photocatalyst applied in each round was identical (35 mg). The mineralization degrees of CIP solution by IBOCH-2 were obtained by detecting the total organic carbon (TOC) on a Shimadzu TOC–LCSH/CPH analyzer.

## 3. Results and Discussion

### 3.1. Characterization

**Figure 1:**
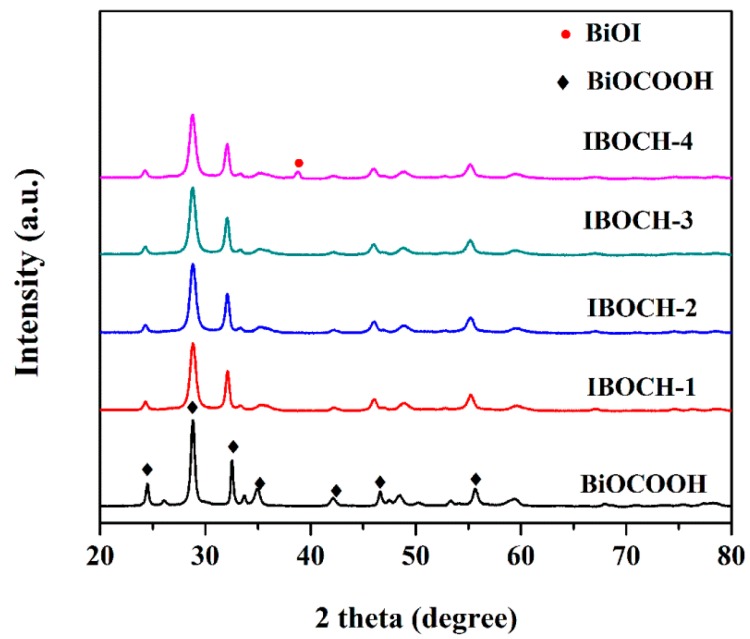
XRD patterns of BiOCOOH, and as-fabricated BiOI/BiOCOOH heterojunctions (IBOCH-1, IBOCH-2, IBOCH-3, and IBOCH-4).

The XRD patterns of all samples are displayed in Figure 1. Pure BiOCOOH was tetragonal in structure (JCPDS No. 35-0939) [13,16]. For IBOCH-1, IBOCH-2, and IBOCH-3 heterojunctions, only the characteristic peaks of BiOCOOH were detected. As the amount of KI was further increased, besides the peaks of BiOCOOH, one diffraction peak indexed to the (004) crystal facet of tetragonal BiOI (JCPDS No. 10-0445) [8,25] was also observed, signifying the presence of the BiOI phase. Moreover, the peaks of other crystals in the XRD pattern were not observed, indicating the good purity of the samples.

**Figure 2:**
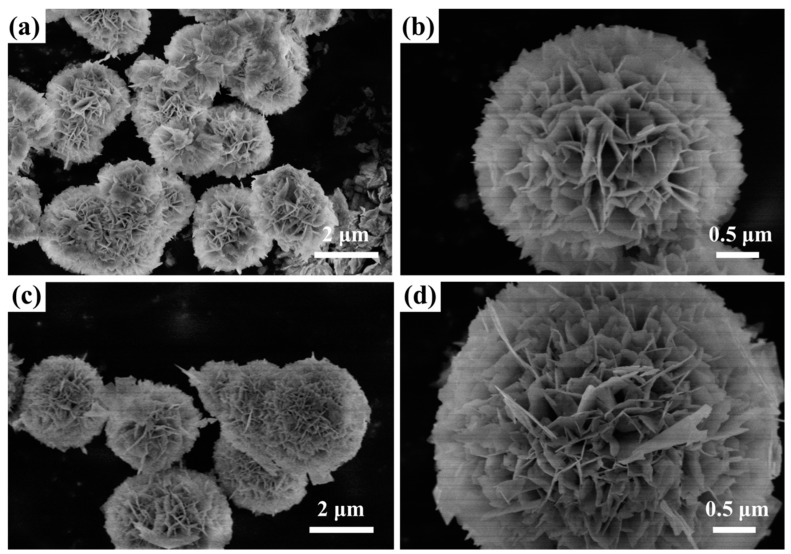
SEM images of (**a**,**b**) BiOCOOH and (**c**,**d**) IBOCH-2.

**Figure 3:**
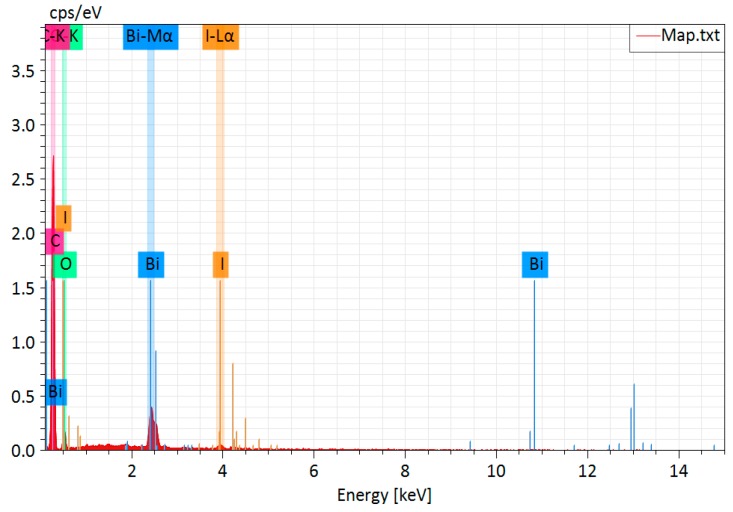
EDX spectrum of IBOCH-2.

Figure 2 displays SEM images of BiOCOOH and the IBOCH-2 heterojunction. It was seen that BiOCOOH microspheres were of flower-like architecture with diameters of 2.0‒4.0 μm (Figure 2a,b). After the anion-exchange treatment, the as-fabricated IBOCH-2 still exhibited a flower-like shape (Figure 2c,d). Moreover, the EDX spectrum (Figure 3) was measured to provide further evidence for the formation of the BiOI/BiOCOOH heterojunction. Apparently, Bi, O, C, and I elements co-existed in IBOCH-2, evidence of the intimate integration between BiOI and BiOCOOH.

**Figure 4:**
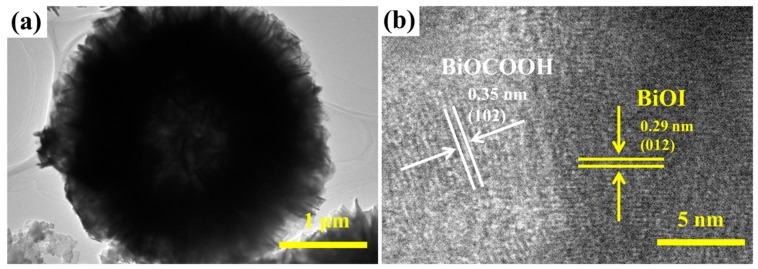
(**a**,**b**) TEM images of IBOCH-2.

The features of IBOCH-2 were further visualized by TEM. As noted in Figure 4a, IBOCH-2 showed a 3D flower-like structure that consisted of 2D nanosheets. The high-resolution TEM (HR-TEM) image of IBOCH-2 (Figure 4b) showed two adjacent lattice fringes with interlayer distances of 0.35 and 0.29 nm, which were associated with the (102) and (012) lattice facets of BiOCOOH and BiOI, respectively. According to the above results, the successful fabrication of BiOCOOH/BiOI heterojunctions can be confirmed.

**Figure 5:**
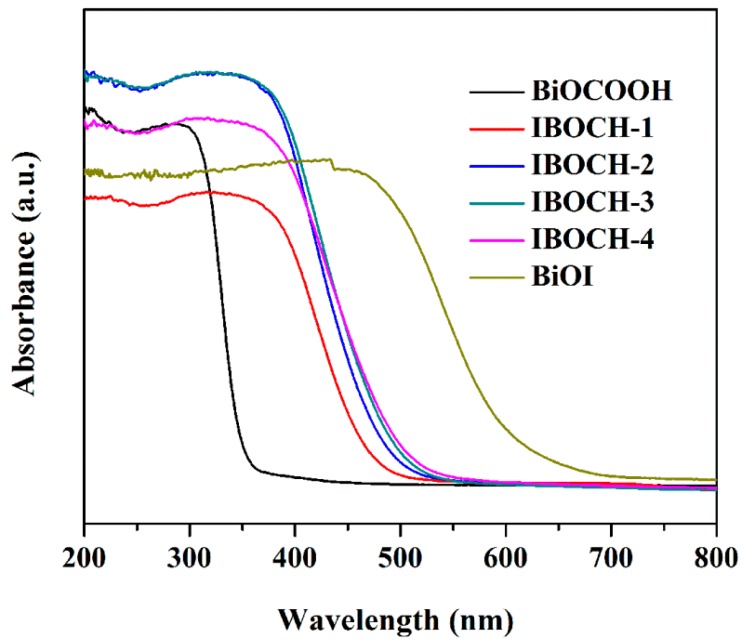
UV‒Vis DRS of the as-fabricated BiOCOOH, BiOI, IBOCH-1, IBOCH-2, IBOCH-3, and IBOCH-4.

UV–Vis spectra of the BiOI, BiOCOOH, and BiOI/BiOCOOH samples were collected to analyze their light-harvesting capabilities (Figure 5). The light-absorption verges of BiOI and BiOCOOH were around 668 (*Eg* = 1.80 eV) [24,25] and 370 nm (*Eg* = 3.40 eV) [14,15], respectively, which are in accordance with the results reported in previous studies. When BiOI was in situ grown on BiOCOOH, the absorption verges of the BiOI/BiOCOOH samples were evidently red-shifted and exhibited remarkably enhanced visible light absorption compared to BiOCOOH, signifying that the as-fabricated heterojunctions could be endowed with high VLD photocatalytic behavior. Further, the band structures of BiOI and BiOCOOH were determined according to the empirical equations

(1) $$E_{VB}\;=\;X\;-\;E_{0}\;+\;0.5E_{g}$$

(2) $$E_{CB}\;=\;E_{VB}\;–\;E_{g}$$

where *E*_VB_, *E*_CB_, *E*_0_, and *X* separately refer to the valence band (VB) potential, conduction band (CB) potential, electronegativity of the semiconductor, and potential energy of free electrons (~4.5 eV). Consequently, the *E*_CB_ and *E*_VB_ for BiOCOOH were calculated as −0.67 and 2.73 eV [16,29], while those for BiOI were determined as 0.54 and 2.34 eV [19,27].

### 3.2. Photocatalytic Properties

**Figure 6:**
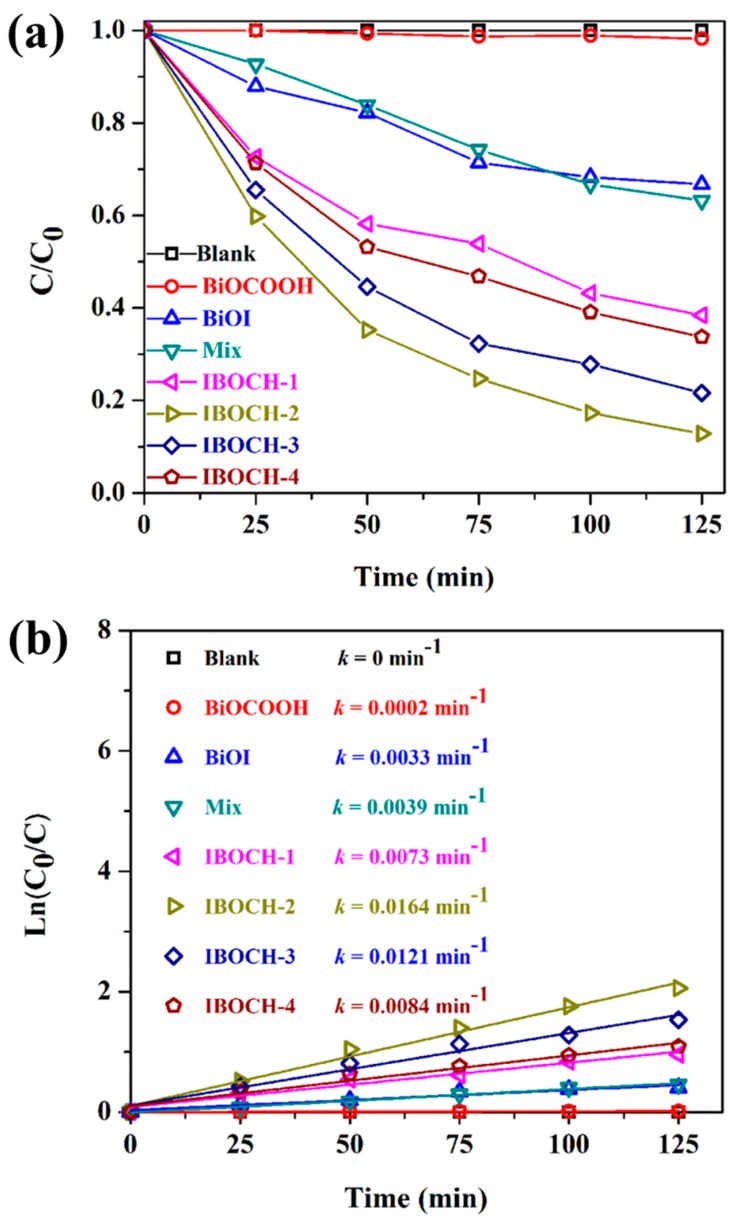
(**a**) Photodegradation of CIP by the as-fabricated samples under visible light; (**b**) Photodegradation kinetics of CIP.

The as-fabricated photocatalysts were utilized to eliminate toxic antibiotics (CIP and TC) under visible light illumination. Figure S1 and Figure 6a present the CIP adsorption and degradation profiles over various samples. Prior to illumination, the mixture containing CIP solution (10 mg/L, 80 mL) and the as-fabricated sample (35 mg) was vigorously agitated in the dark for 30 min to reach the adsorption–desorption equilibrium.

Clearly, no noticeable removal of CIP was detected in the presence of light alone. After 125 min of irradiation, nearly no CIP was degraded by BiOCOOH as its large bandgap made BiOCOOH almost inactive under visible light [12]. Suffering from the rapid recombination rate of carriers [23,30], pure BiOI also showed unsatisfactory photocatalytic performance. Only 33.4% of CIP was removed under the same conditions. Encouragingly, compared to bare BiOI and BiOCOOH, these BiOI/BiOCOOH p–n heterojunctions demonstrated markedly superior photocatalytic behavior, which was probably due to the novel 3D hierarchical heterostructure that triggered the efficient separation of charge carriers. Moreover, the BET surface areas of the as-fabricated materials were tested. As displayed in Table S1, the BET surface areas of BiOCOOH, IBOCH-1, IBOCH-2, IBOCH-3, and IBOCH-4 were 27.35, 29.64, 26.72, 25.28, and 24.83 m^2^·g^−1^, respectively. Though the BET surface area of IBOCH-2 was not the largest among these catalysts, IBOCH-2 demonstrated the optimum catalytic behavior with a CIP degradation efficiency of 87.2% in 125 min. Clearly, the BET surface area was not a vital factor in determining the photocatalytic capability of BiOI/BiOCOOH heterojunctions. Further, the photocatalytic behavior of IBOCH-2 was superior to that of the physically mixed sample (named as mix), highlighting the premier role of a closely contacted interface in determining the activity. Furthermore, for a better understanding of the photocatalytic capability of the as-fabricated catalysts, a kinetic analysis should be performed [31,32]. The pseudo-first-order model was utilized to determine the apparent rate constant (k) of CIP degradation over different samples (Figure 6b). The linearity between ln (C_0_/C) and illumination time (t) was good for all the samples, signifying that the photocatalytic removal of CIP in aqueous solution could be well analyzed by pseudo-first-order reaction dynamics. Of note, the k value using IBOCH-2 was 0.0164 min^−1^, approximately 81- and 3.9-fold greater than using pure BiOCOOH (0.0002 min^‒1^) and BiOI (0.0033 min^−1^), respectively.

**Figure 7:**
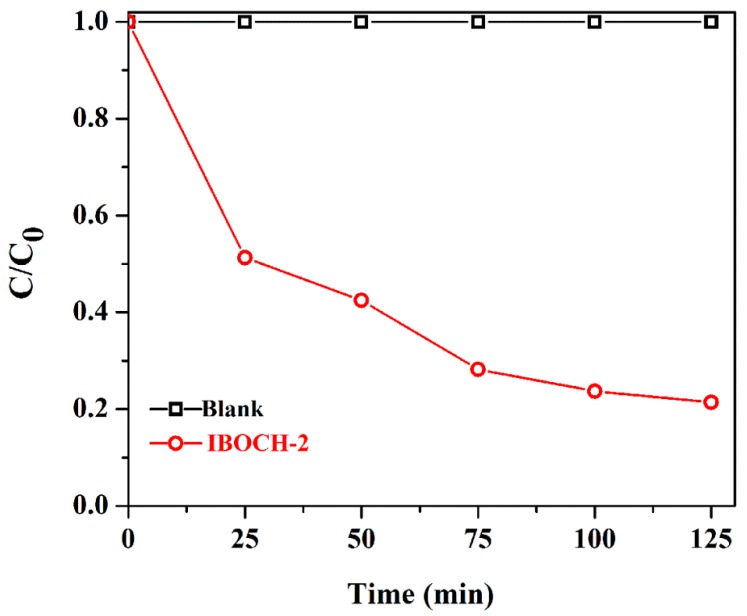
Photodegradation of TC by the IBOCH-2 p–n heterojunction under visible light.

Antibiotic TC, which could induce reproductive abnormalities to people, was used to further demonstrate the excellent photocatalytic behavior of IBOCH-2. As shown in Figure S2 and Figure 7, 78.6% of TC was efficiently removed in 125 min of irradiation, illustrating that IBOCH-2 possessed the high photocatalytic behavior for the elimination of pharmaceutical antibiotics (CIP and TC).

Further, the photocatalytic behaviors of the as-fabricated photocatalysts were examined via the elimination of CIP in water under simulated solar irradiation (Figure S3). Notably, IBOCH-2 also demonstrated the most powerful photocatalytic capability, and 91.4% of CIP was eliminated in 100 min of simulated solar irradiation.

**Figure 8:**
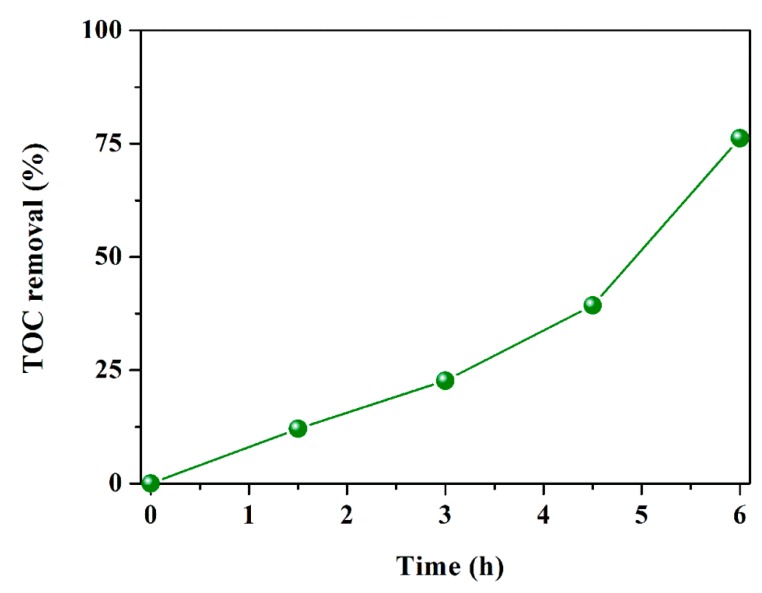
Total organic carbon (TOC) elimination efficiency of CIP over IBOCH-2.

To appraise the mineralization performance of IBOCH-2, the TOC elimination efficiency was determined during the degradation of CIP (40 mg/L, 200 mL) by IBOCH-2 (150 mg). Apparently, IBOCH-2 achieved a remarkable TOC elimination efficiency of 76.2% after 6 h of illumination (Figure 8), illustrating that IBOCH-2 owned remarkable mineralization ability.

**Figure 9:**
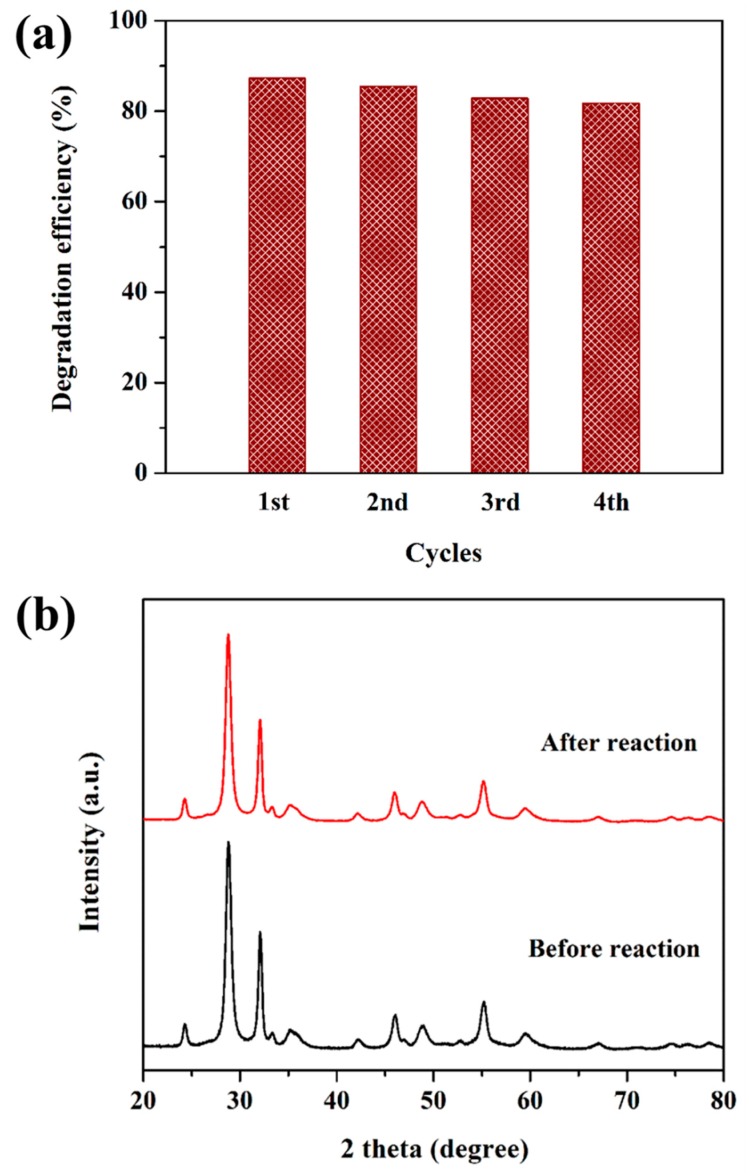
(**a**) Photocatalytic degradation of CIP for four successive runs; (**b**) XRD patterns of IBOCH-2 before and after the recycling test.

The recyclability of the photocatalyst is a premier index for the actual applications [33,34,35]. Therefore, we studied the stability of IBOCH-2 by recycling experiments (Figure 9a). Inspiringly, IBOCH-2 had no appreciable slump (merely 6.3% loss) in photocatalytic capability even after four successive runs. In addition, there were no apparent alternations in its crystalline phases, as evidenced by the XRD analysis of IBOCH-2 before and after the reaction (Figure 9b). The findings verify that IBOCH-2 possesses high activity and good stability. Further, its good stability is probably due to its unique hierarchical heterostructure, which could prevent the photocorrosion of IBOCH-2 by promoting interfacial charge transfer.

### 3.3. Photocatalytic Mechanism

**Figure 10:**
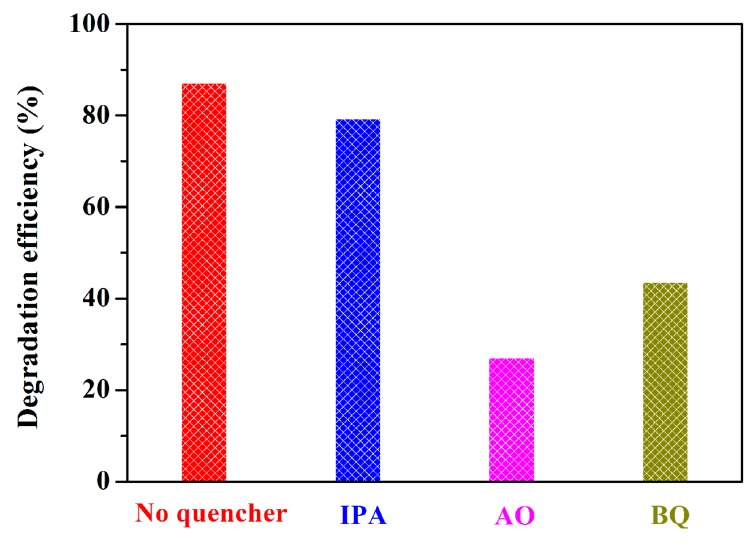
Influences of quenchers on the photocatalytic capability of IBOCH-2.

The roles of different reactive species in the photodegradation of CIP antibiotic was studied via the trapping experiment. As shown in Figure 10, as IPA was added, no significant changes in the photocatalytic activity of IBOCH-2 were observed, suggesting that •OH exerts a secondary impact on CIP removal. However, when BQ and AO were added, the catalytic efficiencies were reduced to 43.6% and 27.1%, respectively, reflecting that superoxide free radicals (•O_2_^−^) and photo-generated hole (h^+^) species have a dominant contribution to the elimination of CIP. In conclusion, •O_2_^−^ and h^+^ species primarily govern the photocatalytic elimination of CIP.

**Figure 11:**
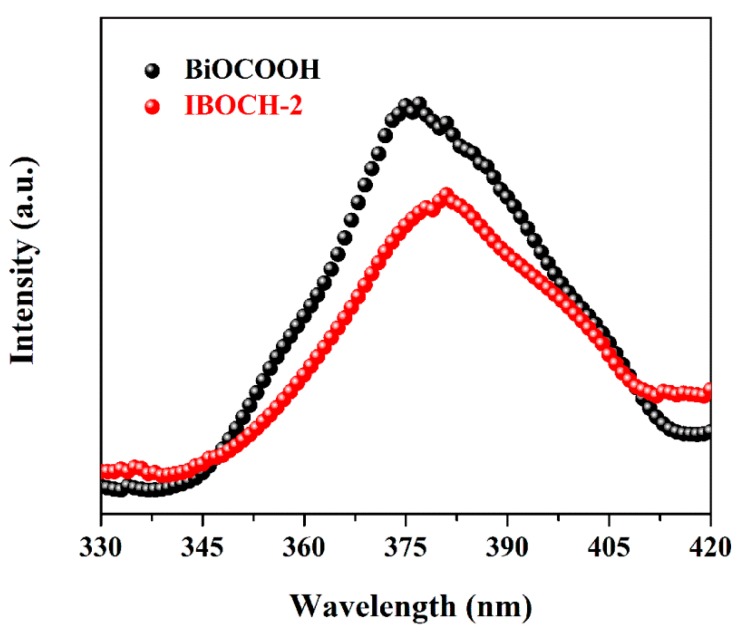
Photoluminescence (PL) spectra of BiOCOOH and the IBOCH-2 heterojunction.

The photoluminescence (PL) spectra can reveal the recombination degree of photo-excited charge carriers, hence, the PL spectra of BiOCOOH and IBOCH-2 were measured (Figure 11). In general, the low PL intensity facilitated charge separation and, consequently, superior photocatalytic ability [30,36,37,38,39,40,41]. Compared to f BiOCOOH, IBOCH-2 exhibited a much lower PL intensity, signifying that the introduction of BiOI can effectively suppress the recombination of charge carriers through promoting the interfacial charge transfer.

**Figure 12:**
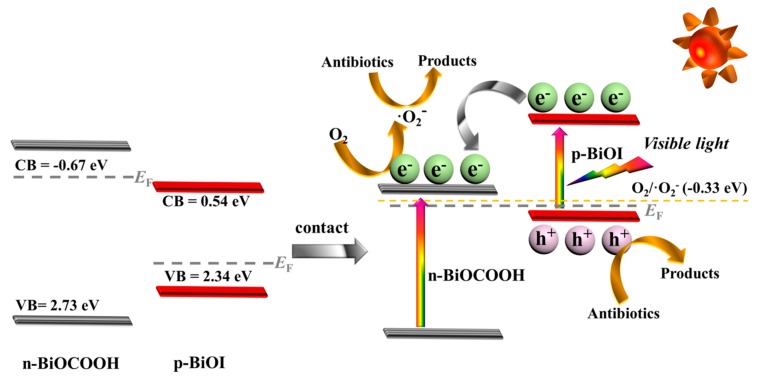
Proposed photocatalytic mechanism of the BiOI/BiOCOOH p–n heterojunction for the elimination of antibiotics under visible light.

On account of the abovementioned characterizations and analyses, a plausible photocatalytic mechanism of BiOI/BiOCOOH p–n heterostructures under visible light was proposed and presented in Figure 12. The VB potentials of BiOCOOH and BiOI were situated at 2.73 and 2.34 eV, respectively, while their corresponding CB potentials were −0.67 and 0.54 eV, respectively. However, when BiOCOOH and BiOI were in contact, a p–n heterostructure was created at the interface. The electrons and holes at the interfaces of the p-type and n-type semiconductors were redistributed to achieve an equilibrium of the Femi energy (*E*_F_). Accordingly, the band bending took place in the space charge area, inducing a strong internal electric field. Of note, such a powerful internal electric field is greatly beneficial to separating the photo-excited electrons and holes [42,43,44]. Simultaneously, the CB and VB positions of BiOI and BiOCOOH shifted along with the movement of *E*_F_. As a consequence, the band position of BiOI was more negative than that of BiOCOOH. Under visible light illumination, the electrons in the VB of BiOI were excited and migrated to the CB, where they rapidly flowed into the CB of BiOCOOH, leaving the photo-excited holes to remain in the VB of BiOI. Such a charge transfer pathway made the separation of photo-excited carriers more effective, contributing to the enhancement of photocatalytic performance [42,43,44]. More specifically, the electrons stored on the CB of BiOCOOH were involved in reacting with O_2_ to generate •O_2_^−^ radicals, which can efficiently degrade antibiotics. On the other hand, the holes remaining on the VB of BiOI were capable of directly eliminating the antibiotics. Under the attack of two crucial reactive species of •O_2_^−^ and h^+^, the antibiotics (CIP and TC) could be effectively removed over the BiOI/BiOCOOH p–n heterojunction under visible light.

## 4. Conclusions

In summary, the hierarchical assembly of BiOI nanosheets embedded in BiOCOOH microflowers with tightly contacted interfaces was achieved via a feasible synthesis for highly efficient pollutant degradation. The resulting BiOI/BiOCOOH heterojunction can offer plenty of charge transfer channels, which could boost the migration and separation of photogenerated charges and, finally, lead to a remarkably improved photocatalytic performance. Specifically, the BIOB-3 heterojunction achieved the highest photocatalytic capacity with an 81-fold photodegradation rate compared to that of BiOCOOH, and 4.9-fold in CIP degradation. Moreover, TOC tests and cycling experiments demonstrated the strong mineralization capability and good stability of BiOI/BiOCOOH. Therefore, heterojunctions are promising for practical wastewater treatment. This study presents a promising route to explore hierarchical heterostructure photocatalysts for environmental purification.

## Supplementary Materials

The following are available online at https://www.mdpi.com/2079-4991/9/11/1571/s1, Figure S1: The absorption profiles of CIP over as-fabricated photocatalysts in the dark. Figure S2: The absorption profiles of TC over as-fabricated IBOCH-2 in the dark. Figure S3: Photodegradation of CIP by the as-fabricated samples under simulated solar irradiation. Table S1: BET surface areas of samples
