# Supplementary material for: Facile Fabrication of Flower-Like BiOI/BiOCOOH p–n Heterojunctions for Highly Efficient Visible-Light-Driven Photocatalytic Removal of Harmful Antibiotics
Source: Nanomaterials (Basel). 2019 Nov 6;9(11):1571. doi: 10.3390/nano9111571 (PMC6915482; doi:10.3390/nano9111571)
Supplement: Supplementary file 1 [file nanomaterials-09-01571-s001.pdf]

nanomaterials

MDPI

Article

Facile Fabrication of Flower-Like BiOI/BiOCOOH p–n Heterojunctions for Highly Efficient Visible-Light-Driven Photocatalytic Removal of Harmful Antibiotics

Shijie Li [1], Bing Xue [1,2], Chunchun Wang [1,2], Wei Jiang [1,*], Shiwei Hu [1,*], Yanping Liu [1,2], Hengwei Wang [1] and Jianshe Liu [3]

[1] Key Laboratory of Health Risk Factors for Seafood of Zhejiang Province, Institute of Innovation & Application, Zhejiang Ocean University, Zhoushan 316022, China. lishijie@zjou.edu.cn (S.L.); xb1725621827@163.com (B.X.); wcc14nb@126.com (C.W.); liuyp@zjou.edu.cn (Y.L.); wanghw@zjou.edu.cn (H.W.)

[2] College of Marine Science and Technology, Zhejiang Ocean University, Zhoushan 316022, China

[3] State Environmental Protection Engineering Center for Pollution Treatment and Control in Textile Industry, College of Environmental Science and Engineering, Donghua University, Shanghai 201620, China. liujianshe@dhu.edu.cn

* Correspondence: jiangwei_zjou@163.com (W.J.); hushiweihai@163.com (S.H.). Tel.: +86-21-67792557 (W.J.)

(Experimental Section).

X-ray diffraction (XRD) data of all as-fabricated catalysts were acquired by a XRD diffractometer (XRD, MiniFlex 600, Rigaku, Japan). The morphological features of all as-fabricated catalysts were observed using a scanning electron microscope (SEM, Hitachi S-4800, Tokyo, Japan) and transmission electron microscope TEM, Tecnai G2F20, Philips, Amsterdam, The Netherlands).. The UV-vis diffuse reflectance spectra (DRS) of all as-fabricated catalysts were measured on a spectrophotometer (Shimadzu UV-2600, Tokyo, Japan). Photoluminescence (PL) analyses were conducted on a spectrophotometer (Hitachi F-7000, Tokyo, Japan).

Table S1. BET surface areas of samples.

Samples	BiOCOOH	IBOCH-1	IBOCH-2	IBOCH-3	IBOCH-4
BET($m^2 \cdot g^{-1}$)	27.35	29.64	26.72	25.28	24.83

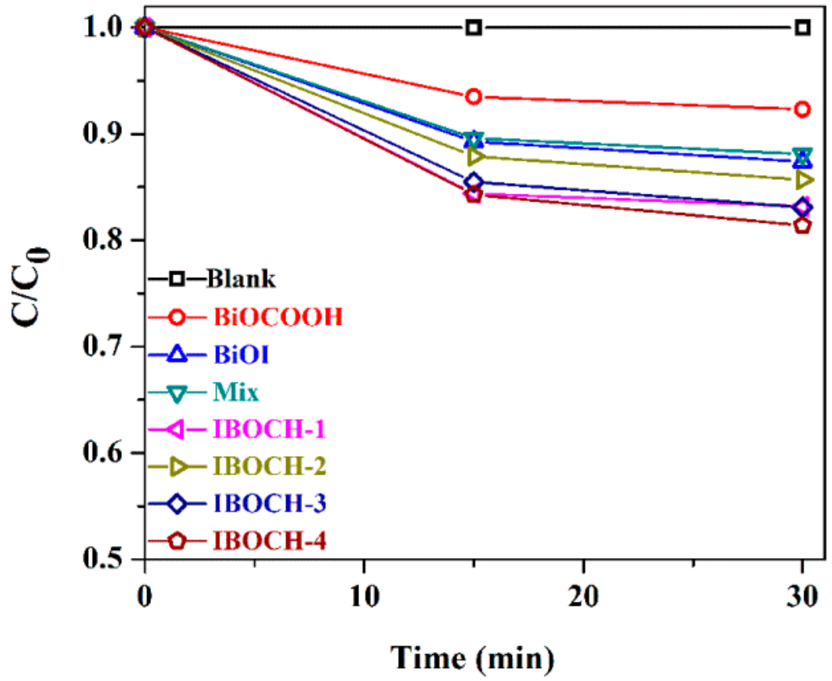

Figure S1. The absorption profiles of CIP over as-fabricated photocatalysts in the dark.

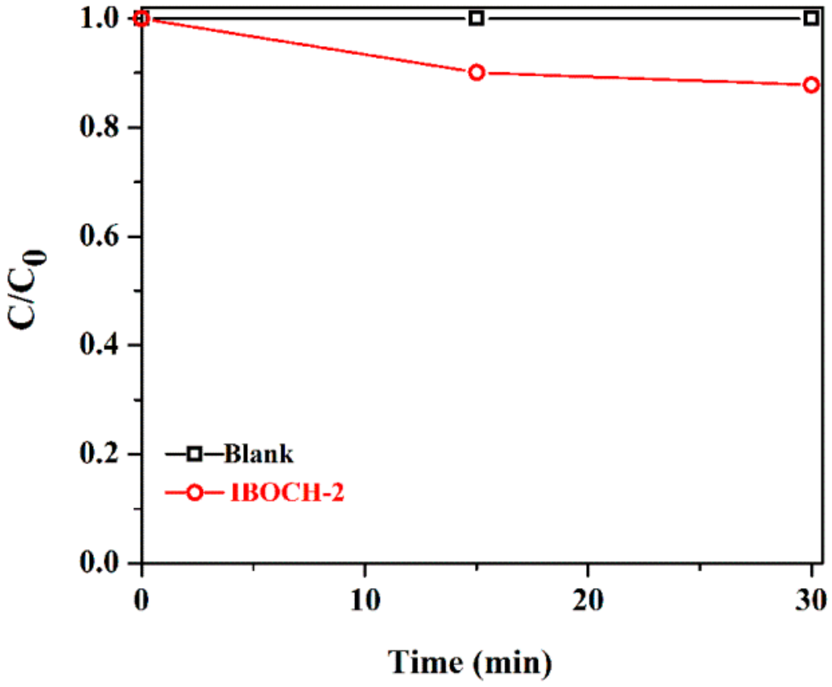

Figure S2. The absorption profiles of TC over as-fabricated IBOCH-2 in the dark.

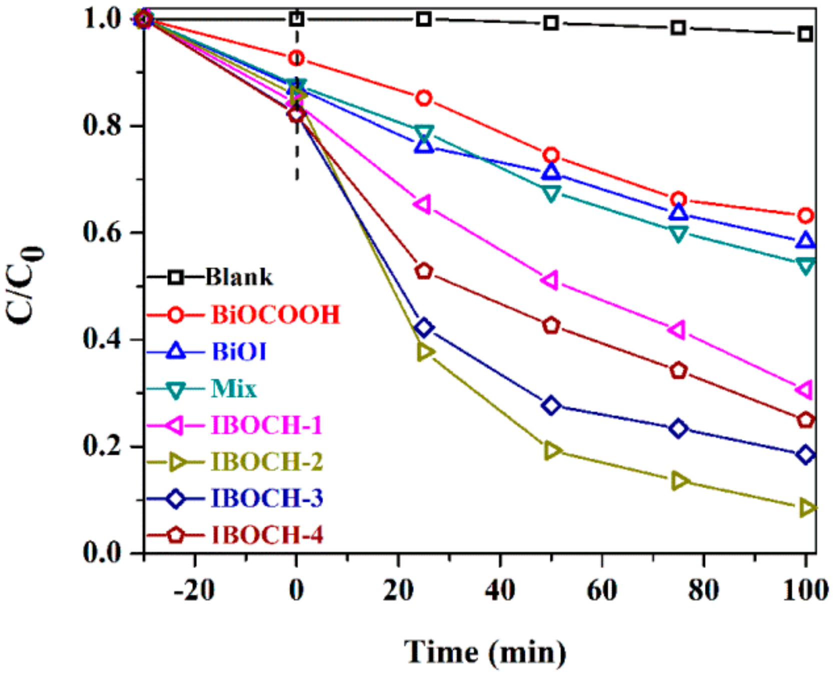

Figure S3. Photodegradation of CIP by the as-fabricated samples under simulated solar irradiation.

© 2019 by the authors. Submitted for possible open access publication under the terms and conditions of the Creative Commons Attribution (CC BY) license (http://creativecommons.org/licenses/by/4.0/).
